# Anion-Transport Mechanism of a Triazole-Bearing Derivative of Prodigiosine: A Candidate for Cystic Fibrosis Therapy

**DOI:** 10.3389/fphar.2018.00852

**Published:** 2018-08-07

**Authors:** Claudia Cossu, Michele Fiore, Debora Baroni, Valeria Capurro, Emanuela Caci, Maria Garcia-Valverde, Roberto Quesada, Oscar Moran

**Affiliations:** ^1^Istituto di Biofisica, Consiglio Nazionale Delle Ricerche, Genova, Italy; ^2^U.O.C. Genetica Medica, Istituto Giannina Gaslini, Genova, Italy; ^3^Departamento de Química, Facultad de Ciencias, Universidad de Burgos, Burgos, Spain

**Keywords:** cystic fibrosis, ionophore, ion transport, phospholipid vesicles, prodigiosin derivatives

## Abstract

Cystic fibrosis (CF) is a genetic lethal disease, originated from the defective function of the CFTR protein, a chloride and bicarbonate permeable transmembrane channel. CF mutations affect CFTR protein through a variety of molecular mechanisms which result in different functional defects. Current therapeutic approaches are targeted to specific groups of patients that share a common functional defect. We seek to develop an innovative therapeutic approach for the treatment of CF using anionophores, small molecules that facilitate the transmembrane transport of anions. We have characterized the anion transport mechanism of a synthetic molecule based on the structure of prodigiosine, a red pigment produced by bacteria. Anionophore-driven chloride efflux from large unilamellar vesicles is consistent with activity of an uniporter carrier that facilitates the transport of anions through lipid membranes down the electrochemical gradient. There are no evidences of transport coupling with protons. The selectivity sequence of the prodigiosin inspired EH160 ionophore is formate > acetate > nitrate > chloride > bicarbonate. Sulfate, phosphate, aspartate, isothionate, and gluconate are not significantly transported by these anionophores. Protonation at acidic pH is important for the transport capacity of the anionophore. This prodigiosin derived ionophore induces anion transport in living cells. Its low toxicity and capacity to transport chloride and bicarbonate, when applied at low concentration, constitute a promising starting point for the development of drug candidates for CF therapy.

## Introduction

Cystic fibrosis (CF), the most common autosomal recessive lethal genetic disease in the Caucasian population (Strausbaugh and Davis, 2007), is caused by mutations on the gene coding for CFTR (cystic fibrosis transmembrane conductance regulator), an anion selective channel that transports chloride and bicarbonate in the apical membrane of epithelium. CFTR regulates salt and water transport across a variety of epithelium, and malfunction of this protein leads to defective mucus and airway surface liquid (ASL) properties, resulting in poor mucus clearance and bacterial infections on the airways (Berger et al., 1991; Saint-Criq and Gray, 2017). More than 1,500 mutations in CFTR, classified in six classes according to the effect of the mutation, lead to anion transport defects in epithelium by various mechanisms (failure to synthesize the protein, processing flaws, gating or conductance defects, reduced expression). In cell models, several small molecules, called correctors, increase CFTR defective expression at the cell membrane, and small molecules called potentiators to enhance the function of the CFTR channel (Zegarra-Moran and Galietta, 2017). While the potentiator ivacaftor (Kalydeco™) is successfully used for treatment in patients with gating mutations (Ramsey et al., 2011; De Boeck and Davies, 2017), the corrector lumacaftor, alone or in the combination with ivacaftor (Orkambi™) resulted on a limited clinical outcome (Clancy et al., 2012; Wainwright et al., 2015).

The discovery of small molecules capable to transport anions across lipid bilayers, named anionophores, has offered the opportunity of designing new substances to be used for pharmacological purposes (Davis et al., 2010; Valkenier et al., 2014; Hernando et al., 2018). The most striking among the potential applications of anionophores is the proposal to use these substances to substitute the defective anion transport in cystic fibrosis (CF). The idea of using anionophores to replace the defective CFTR protein has the advantage to become a general therapy for CF, that would be independent of the specific mutation. Different chemical structures have been described to transport anions across lipid bilayers, including prodigiosin and obatoclax derivatives (Seganish and Davis, 2005; Díaz de Greñu et al., 2011; García-Valverde et al., 2012; Gale et al., 2013), marine alkaloids such as tambjamines (Iglesias Hernández et al., 2012; Saggiomo et al., 2012; Hernando et al., 2014; Soto-Cerrato et al., 2015), steroid-based “cholapods” (Koulov et al., 2003; McNally et al., 2008; Valkenier et al., 2014), and calix[4]pyrrole derivatives (Gale et al., 2017), anion channel-forming peptides (Wallace et al., 2000; Broughman et al., 2004; Pajewski et al., 2006) and Calix[4]arene amides (Sidorov et al., 2002).

We have previously identified several triazole derivatives of prodigiosin with a significant anion transport capacity and relatively low toxicity (Hernando et al., 2018). There, we show that these compounds can transport chloride and bicarbonate across lipid bilayers, and the transport activity increases at acidic pH. Also evidences of anionophore-driven halides transport in cells is provided. Hence, to follow the search of “druggable” anionophores adequate for CF therapy, we have undertaken the analysis of the transmembrane anion transport mechanism of these molecules. Thus, for the functional characterization of this anionophore family, we chose of the most active substance of that series, EH160 (named **1b** in reference Hernando et al., 2018). Here, we have extended the data previously reported (Hernando et al., 2018), analysing the selectivity of the anionophore, demonstrating that the carrier has a good selectivity for physiologically relevant anions, such as chloride and bicarbonate. An important finding was that pH influence on the ionization state determines the anionophore activity, without any proton transport. We showed that this prodigiosin-inspired anionophore act as electro-neutral anion exchanger. To reinforce the concept that anionophores can induce chloride transport in mammalian cells, we repeated chloride efflux and iodide influx experiments in cells, similar to those reported elsewhere (Hernando et al., 2018).

## Materials and methods

### Synthesis of the anionophore EH160

EH160 was synthesized as previously described (Hernando et al., 2018). In brief, it was prepared by acid-catalyzed condensation of 3-methoxy-5-(1-butyl-1H-1,2,3-triazol-4-yl)-1H-pyrrole-2-carbaldehyde and 2-methyl-3-pentyl-1H-pyrrole. The precursor aldehyde was prepared by standard click chemistry reaction between 1-azidobutane and the corresponding 5-ethynylpyrrole carbaldehyde. The compound is inspired by the structure of natural products prodiginines, replacing one of the pyrrole groups by a 1,2,3-triazole moiety. EH160 was fully characterized by mass spectroscopy and NMR (Hernando et al., 2018). Except when indicated, all chemicals were purchased from Sigma-Aldrich.

### Large unilamellar vesicles

Asolectin large unilamellar vesicles (LUV) were made from phospholipids films (Baroni et al., 2014; Nicastro et al., 2016; Hernando et al., 2018). Soybean phospholipids (20 mg/ml) were dissolved in chloroform and lipid films were obtained by evaporation of the solvent under a gentle nitrogen flux; in order to remove all chloroform, films were further dried overnight in vacuum. The phospholipids were hydrated in chloride buffer (in mM: NaCl 450, 20 mM HEPES; pH 7.5, unless other composition was indicated), and vigorously vortex mixed and, to ensure equilibration, sonicated in 5 cycles of 1.5 min each, with 1 min rest, in ice. Liposomes were centrifuged at 2,000 g for 5–10 min to remove any titanium particles released by the sonicator tip. Large unilamellar vesicles (LUV) were then obtained by extrusion through polycarbonate filters mounted in a mini-extruder (Lipofast, Avestin, Mannheim, Germany). Samples were subjected to 19 passes through a single 100 nm mesh filter (MacDonald et al., 1991). External solution was exchanged twice on a Sephadex G25 column previously equilibrated with the external chloride-free solution: NaNO_3_ 450 mM, 20 mM HEPES; pH 7.5 (unless other composition was indicated).

### Chloride efflux measurements in LUV

To measure the efflux of chloride from LUV, chloride concentration was measured with an ion-sensitive electrode (Vernier, Beaverton, Oregon, USA) in a constantly stirred 3.5 ml LUV suspension. Data were acquired using a LabQuest mini interface (Vernier). Ionophores were dissolved in DMSO to a concentration of 10 mM. After an initial equilibration, chloride efflux was induced by a small volume (<1%) of ionophore. Control experiments where similar amounts of DMSO (without anionophores) were added demonstrated that these concentrations of DMSO do not induce any chloride efflux (see below). The measurement was concluded with the addition of the detergent polyoxyethylene 10 tridecyl ether (C13E10) to break off the bilayers and measure the maximum chloride encapsulated in the LUV. The time course of the chloride concentration, *Cl*, in the experiment can be described by a single exponential function:

(1) $$\begin{array}{l} Cl^{-}\left(t\right)=Cl_{0}^{-}+\Delta Cl^{-}\left(1-\text{exp}\left[-tk\right]\right) \end{array}$$

where ${\text{Cl}}_{0}^{-}$ is the initial chloride concentration, Δ*Cl*^−^ is the maximum change of the chloride concentration (after addition of detergent), and *k* is the rate constant of the process. The chloride efflux, *J*_*cl*_, is defined as the time derivative of *Cl*^−^(*t*). Thus, deriving from equation (1), for *t* = 0, i.e., after the chloride gradient was changed, we obtain the initial chloride efflux-rate, *J*_0_:

(2) $$\begin{array}{l} J_{0}=\Delta Cl^{-}k \end{array}$$

To compare different data sets, the chloride concentration was normalized to the maximum concentration change, Δ*Cl*^−^. Data are expressed as means ± s.e.m. Experiments were done at 25 ± 1°C. Every experimental condition was repeated at least three times.

### Cell preparation and viability

The Human embryonic kidney (HEK) cell lines were grown in standard conditions, in Ham's F10 medium supplemented with 2 mM L-glutamine and 10% fetal bovine serum (FBS). Cell toxicity was evaluated by the trypan blue exclusion staining method (Louis and Siegel, 2011). Toxicity data is expressed as the half maximum toxic dose (TD_50_).

### Chloride efflux in cells

Cells at 80% confluence were detached from the bottom of the flask by soft scrapping, washed in chloride-free solution, and used immediately. For chloride efflux measurement, ~2 × 10^6^ cells were suspended in 4 ml of buffer containing (in mM): 136 NaNO_3_, 3 KNO_3_, 2 Ca(NO_3_)_2_, 20 HEPES, 11 Glucose, pH 7.4. Ionophores were dissolved in DMSO to a concentration of 10 mM. Chloride concentration in the extracellular solution was continuously measured with a chloride-sensitive electrode. After an initial equilibration, chloride efflux was induced by a small volume (<1%) of ionophore. The measurement was concluded with the addition of the sodium dodecyl sulfate (SDS) to break off the membranes and measure the total chloride content in the cells. Experiments were done at 25 ± 1°C.

### Iodine influx in cells

The activity of anionophores was determined in Fisher Rat Thyroid (FRT) cells expressing the halide-sensitive YFP protein as previously described (Caci et al., 2008). The assay is based in the fact that the fluorescence of the YFP protein is quenched to a greater extend by I^−^ than by Cl^−^ (Galietta et al., 2001). FRT cells stably transfected with a halide-sensitive yellow fluorescent protein (YFP-H148Q/I152L) were plated on 96-well micro-plates at a density of 40,000 cells/well in Coon's modified medium supplemented with 10% serum, 2 mM L-glutamine, 1 mg/ml penicillin, 100 μg/ml streptomycin, and 0.5 mg/ml hygromycin as selection agent for the YFP. Cells were maintained at 37°C in a 5% CO_2_ /95% air atmosphere. Functional experiments were done 48 h after cell seeding. Cells were washed twice, with an external solution containing (in mM): NaCl 137, KCl 2.7, Na_2_HPO_4_ 8.1, KH_2_PO_4_ 1.5, CaCl_2_ 1 and MgCl_2_ 0.5 (pH 7.3). The solution injected during the assay is similar but contained NaI 137 mM instead of NaCl (pH 7.3). To explore the effect of lowering the extracellular pH, the NaI solution was buffered at pH 6.9 with HEPES, or at pH 6.6 and 6.2 using MES. For the iodide influx assay, after washing, cells were incubated in 60 μl of the 137 mM NaCl-external solution, supplemented with the anionophore or with DMSO as control. A fluorescence baseline was recorded for 2 s after injection of 165 μl of NaI-external solution, so that the final concentration of NaI in the well is 100 mM. The iodide influx was observed as quenching of the YFP fluorescence.

## Results

**Figure 1:**
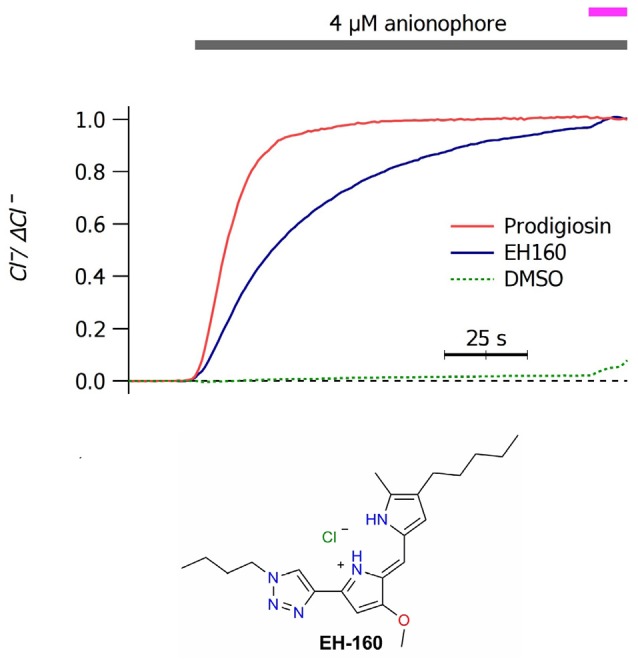
Time course of the external concentration of chloride in a LUV suspension upon application of 4 μM of prodigiosin and EH160, as indicated by the upper bar. The magenta bar indicates the addition of detergent. A control trace was obtained after the application of DMSO. Data was normalized by the maximum chloride change, Δ*Cl*^−^. The chemical structure of EH160 is shown at the bottom.

Application of micromolar concentrations of anionophores to a LUV suspension induces a chloride efflux. In Figure 1A we show the time course of chloride efflux measured upon the application of 4 μM of prodigiosine and the triazole derivative EH160. The natural product, prodigiosine, is a more effective transporter, with a *J*_0_ of 110.2 ± 1.2 μM/s. Instead, the triazole derivative EH160 induces a slower chloride efflux of 51.8 ± 0.5 μM/s. Application of DMSO does not induce a significant chloride efflux (*J*_0_ of 0.2 ± 2.8 μM/s), confirming that the anion efflux was elicited by the anionophore and not by the solvent. Similar experiments were done using LUV formed with chemically defined phospholipids (palmitoyl-oleyl-phosphatidylcholine, POPC; palmitoyl-oleyl-phosphatidylethanolamine, POPE) and cholesterol (chol). Anionophore-driven chloride efflux measured in LUV composed by POPC:chol (19:1) (48.9 ± 0.4 μM/s), POPC:POPE (9:1) (52.3 ± 0.2 μM/s) and POPC:POPE:chol (8.1:0.9:1) (47.1 ± 0.9 μM/s), was very similar to that measured in asolectin LUV, indicating that the lipid composition is not critical for the anionophore transport (data not shown). Similarly, substitution of sodium by potassium in the internal or in the external solutions does not affect the measured chloride efflux, confirming that EH160 is an anion selective carrier.

**Figure 2:**
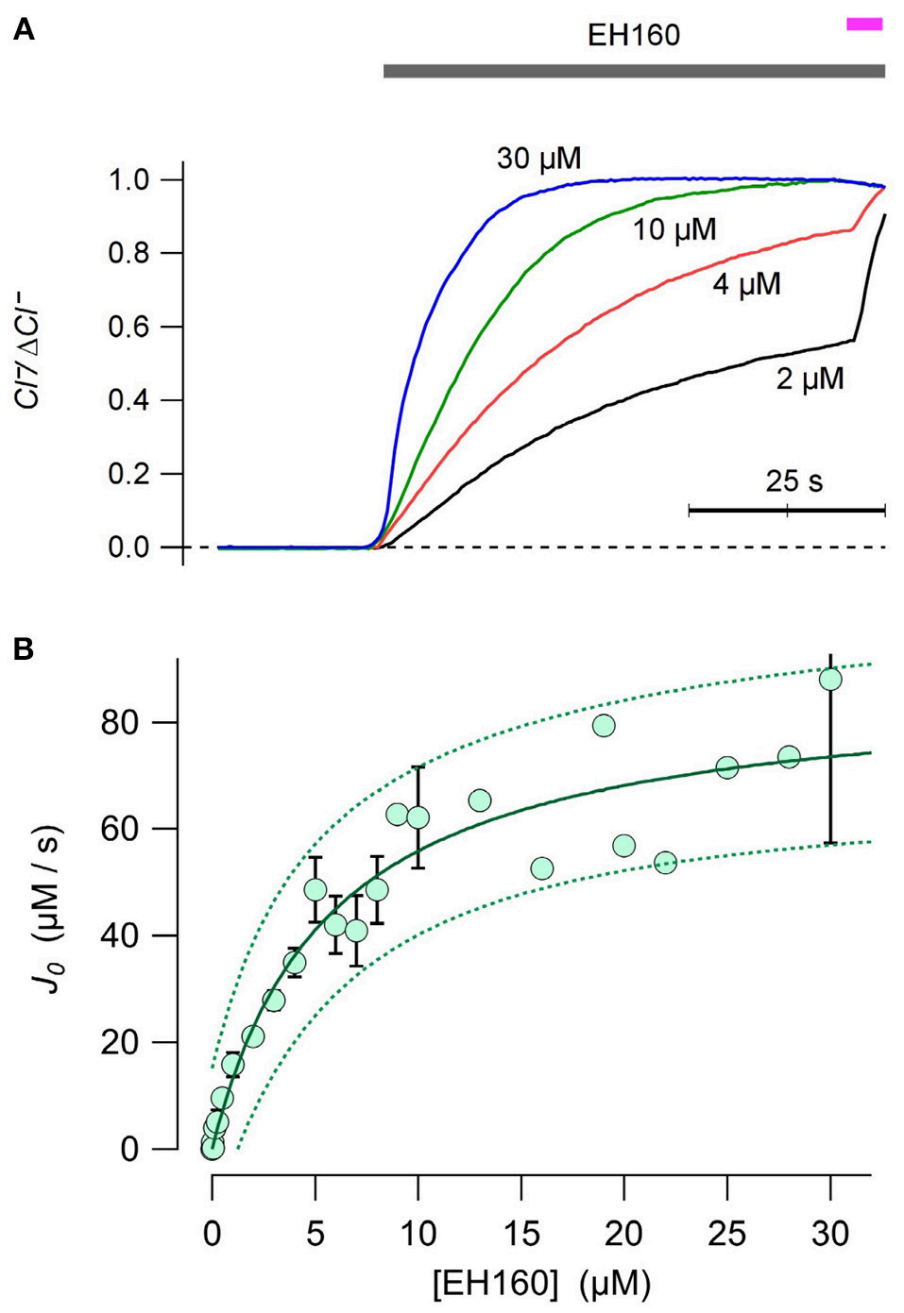
**(A)** Time course of external concentration of chloride elicited by various concentrations of EH160, as shown near each trace. Application of the anionophore is indicated by the upper horizontal bar, and the addition of detergent is indicated by the magenta bar. Data was normalized by the maximum anion change, Δ*Cl*^−^. **(B)** The initial chloride efflux rate, *J*_0_ is plotted against the anionophore concentration. When the measurement was repeated more than once, data represent the average and the bar is the standard error of the mean. The continuous line is the best fit of data with equation 3, yielding the concentration for the half of the maximum effect, EC_50_ = 5.6 μM, and a maximum chloride initial efflux rate of 87.3 μmoles/s. Broken lines represent the prediction interval for 95% confidence.

The chloride efflux rate depends on the concentration of anionophore (see Figure 2A). To evaluate the concentration to induce half of the maximum rate, EC_50_, was estimated plotting the initial efflux rate, *J*_0_, against the anionophore concentration, [EH160] (Figure 2B). Data was fitted with the Equation (3):

(3) $$\begin{array}{l} J_{0}=\frac{J_{max}}{\text{1+}\frac{\;\;\;{\text{EC}}_{50}}{\left[\text{EH160}\right]}} \end{array}$$

where *Jmax* is the maximum chloride efflux initial rate. The time course of the traces obtained at EH160 concentration higher than 10 μM was often quite variable. We interpreted these data as a destabilization of the LUV bilayers. Thus, to remove these possible outliers, we fitted iteratively the data, removing data points that lie beyond the 95% confidence prediction interval at each iteration, until no outliers remain. After this procedure, the doses-response fit yielded a maximum initial chloride efflux of 87.3 ± 6.4 μM/s, and an EC_50_ of 5.64 ± 1.28 μM.

### Selectivity of the anionophore

**Figure 3:**
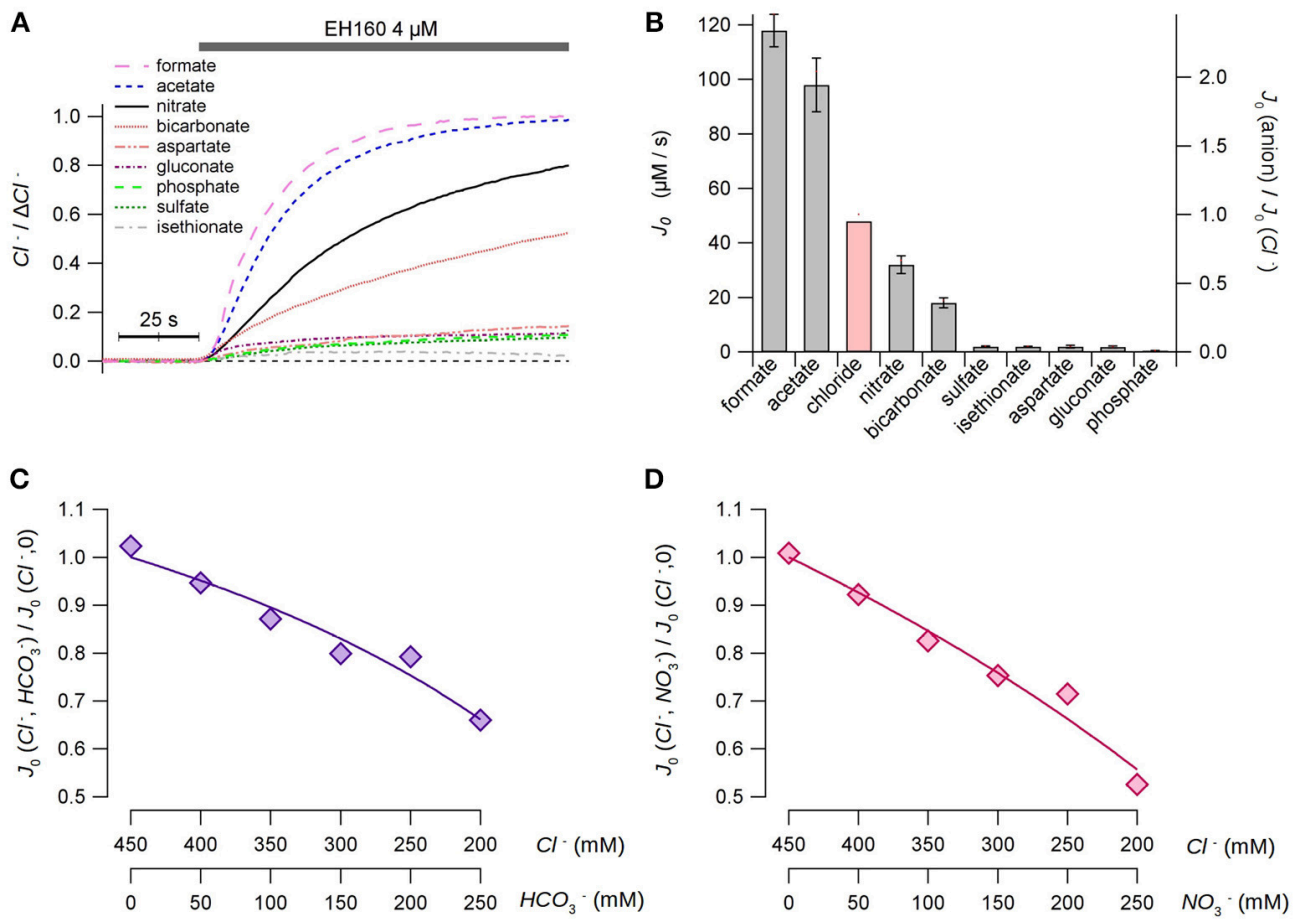
Permeability of the anionophore EH160 to different anions. **(A)** Time course of the external concentration of chloride measured in LUV with 450 mM internal chloride and variable external iso-osmotic anions, as indicated in the figure. The application of the anionophore is indicated in the upper bar. Data was normalized by the maximum anion change, Δ*Cl*^−^. **(B)** The initial chloride efflux, *J*_0_, measured for different external anions. The red bar is the estimated chloride efflux in the presence of external chloride, calculated from the apparent dissociation constants of the anionophore for chloride, bicarbonate and nitrate estimates in the competition experiments. Data represent the average (± s.e.m.) of, at least, three different measurements. Panels **(C,D)** show the results of the bicarbonate/chloride and nitrate/chloride competition experiments, respectively. The concentrations of anions are shown in the bottom axes of the figures. The continuous lines represent the best fit of data with Equation (4).

To evaluate the selectivity of the anionophores, we measured the chloride efflux from LUV with an internal concentration of 450 mM chloride, and the external solution containing an isomolar concentration of different anions. Measurements were done in the presence of 10 mM HEPES to adjust the pH at 7.5 in both compartments. As no differences in the chloride efflux were observed substituting sodium by potassium, neither, in the internal solution nor in the external solutions, the cationic ion was used indifferently in this series of experiments. The time course of the chloride efflux measured with different external anions is shown in Figure 3A.

As a first approach, we could hypothesize that the anionophore interchanges the anions from both sides of the bilayer, and the chloride efflux is proportional to the efficiency of the counter-anion influx. Thus, the estimation of the initial rate of the chloride efflux should represent the permeability of the external anion. From these data (Figure 3B), we can assert that the anionophore EH160 has a consistent transport rate for small organic anions, as acetate and formate, and in less extend for inorganic anions, as nitrate and bicarbonate. Conversely, transport of bigger, more hydrophilic anions, as aspartate, gluconate, phosphate, sulfate, and isethionate, is more than ten-fold reduced.

However, in this experimental design it is not possible to use external chloride to compare the relative permeability of this anion with other anions. To overcome this limitation we designed a series of experiments in which a fraction of the internal chloride was substituted by nitrate or bicarbonate. Thus, the second anion will compete with chloride for binding the anionophore, and therefore, the chloride efflux will be modified accordingly. The initial chloride efflux rates, measured for different combinations of chloride/nitrate and chloride/bicarbonate at the internal side are shown in Figures 3C,D, respectively. The reduction of the chloride efflux, as the concentration of the second anion increases, occurs because the carrier binds bicarbonate or nitrate instead of chloride, resembling a competitive inhibition of an enzyme. Hence, the continuous lines in Figures 3C,D represent the best fits of data with:

(4) $$\begin{array}{l} \frac{J_{0}\left(Cl^{-},anion\right)}{J_{0}\left(Cl^{-},0\right)}=\frac{Cl^{-}}{K_{Cl}\left(1+\frac{anion}{K_{anion}}\right)+Cl^{-}} \end{array}$$

where *K*_*cl*_ and *K*_*anion*_ are the apparent dissociation constants of chloride and the second anion (nitrate or bicarbonate), respectively. The average chloride apparent dissociation constant is *K*_*cl*_ = 3.17 ± 0.48 mM, and the apparent dissociation constants for nitrate and bicarbonate are *K*_*N*_*O*__3__ = 4.79 ± 0.67 mM and *K*_*HC*_*O*__3__ = 8.18 ± 0.71 mM. The higher affinity of chloride (lower apparent dissociation constant) clearly reflects a higher permeability of this ion. Thus, we can assume that the ratio of the apparent dissociation constants *K*_*N*_*O*__3__/*K*_*HC*_*O*__3__ = 0.59 is proportional to the bicarbonate:nitrate permeability ratio. This concept is confirmed by the ratio of the initial efflux rates of 0.57, measured with bicarbonate and nitrate in the external solution (Figure 3A). These data allow to estimate the hypothetical chloride initial efflux rate when the external solution is chloride, yielding a value of 48 μM/s. Scaling data to the theoretical chloride initial efflux rate it is possible to estimate the relative permeability of all assayed ions with respect of chloride, as shown in the right axis in Figure 3B.

**Figure 4:**
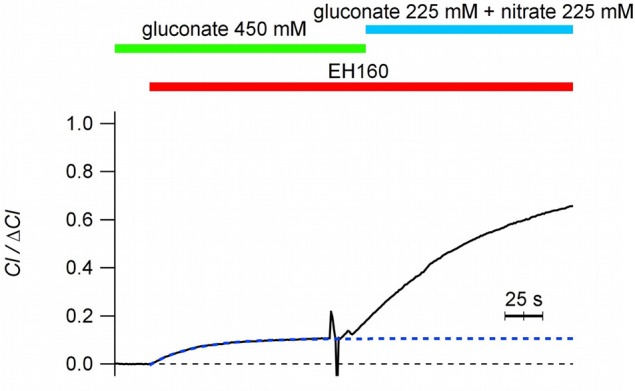
Time course of the external concentration of chloride induced by the anionophore EH160 with different external anions, as indicated in the figure. When gluconate is in the external solution, chloride efflux is small, and is arrested without reaching the equilibria of concentrations (internal *Cl* = 49.5 mM; external *Cl* = 0.099 mM). Further addition of nitrate, a permeable anion, restores the normal anionophore-induced chloride efflux. Data was normalized by the maximum anion change, Δ*Cl*^−^.

It is intriguing to notice that when nominally impermeable anions, such as sulfate and gluconate, are in the external solution, a tiny chloride efflux induced by anionophores is still measurable, although it is very small (chloride efflux of 1.96 and 1.75 μM/s for sulfate and gluconate, respectively). As previously reported (Hernando et al., 2018), in these cases, after the initial chloride efflux, the system seems to stop transport chloride, and no further changes in the external chloride concentration are measured by the ion sensitive electrode (see Figure 4). The trace in Figure 4 show the time course of the external chloride concentration upon the addition of 8 μM EH160 in a suspension of LUV with the internal solution containing 450 mM chloride and the external solution of 450 μM gluconate. The initial chloride efflux is 3.4 μM/s, and the flux is arrested when the external chloride concentration is 0.11 × Δ*Cl*. The successive addition of a permeable anion to the external solution, to a final concentration of 225 mM of nitrate, induces again a chloride efflux. The restarted efflux, *J*_0_ = 6.2 μM/s, is larger than the efflux obtained with only gluconate in the external solution, even after the 11% reduction of the chloride gradient and the dilution of the anionophore. In the presence of external impermeable anions, the initial chloride efflux occurs until the net charge displacement, occurring when the chloride ions move outside from the LUV, is balanced by the potential difference because of the asymmetrical distribution of permeable ions.

**Figure 5:**
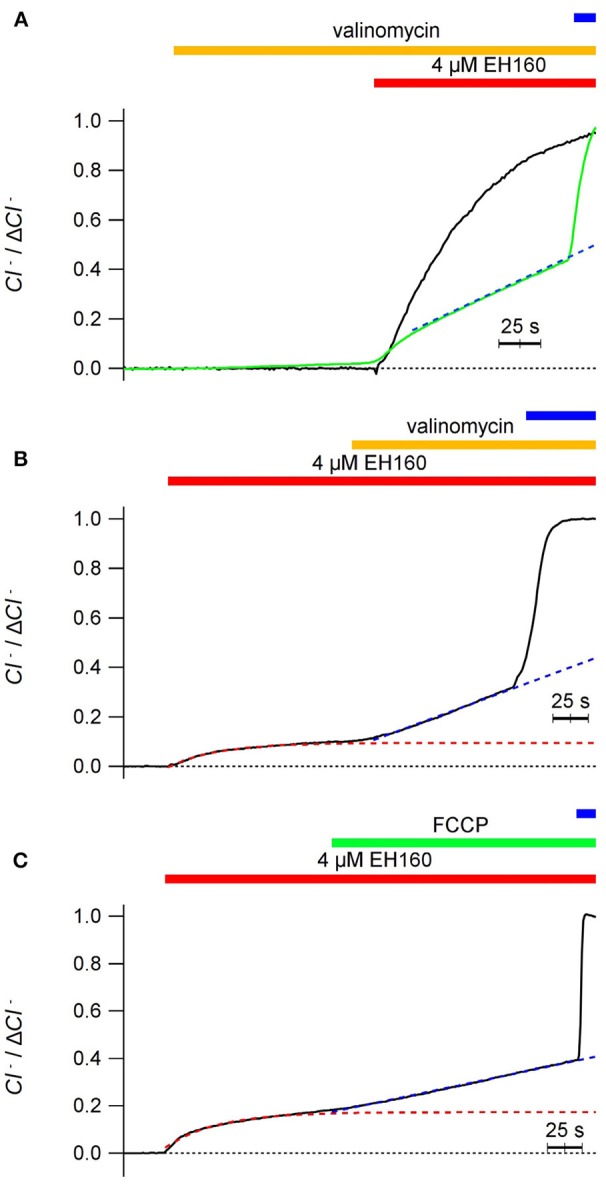
Time course of the external concentration of chloride, revealing the anion efflux induced by the anionophore EH160 recorded in LUV shunted with a second ionophore. In all cases LUV had 450 mM internal potassium chloride and the application of 4 μM of EH160 is indicated by the horizontal (red) bar over the traces. Application of 0.5 ng/ml of valinomycin or 0.25 μg/ml of FCCP is indicated by the middle horizontal bar. The upper horizontal bar indicate the addition of detergent. Data was normalized by the maximum anion change, Δ*Cl*^−^. In panel **(A)**, the anionophore was added after the application of valinomicyn in preparations with the external solution containing 450 mM potassium nitrate (black trace) and 450 mM potassium aspartate (green trace); the external solution in panel **(B)** was 300 mM of potassium sulfate, and in panel **(C)** was 450 mM potassium gluconate. The blue broken lines are the lineal fitting of the traces after application of the cation ionophores. The red broken lines in panels **(B,C)** are the exponential fitting of the traces before the application of the cation ionophores.

Thus, to avoid the charge accumulation and be independent of the potential difference, we “shunted” the LUV bilayer with cationic carriers. The addition of valinomycin (0.5 ng/ml), a specific potassium transporter, does not induce any chloride transport in the vesicles (Figure 5A), and does not modify the typical chloride efflux induced by the anionophore when the external ion is permeable. Differently, in the presence of an impermeable anion, as sulfate, addition of valinomycin restore the chloride efflux (Figure 5B). The same result, reactivation of the chloride efflux halted by an impermeable anion, gluconate outside, is obtained by the addition of 25 μg/ml of carbonyl cyanide-4-phenylhydrazone (FCCP), a proton ionophore (Figure 5C). The application of valinomycin before the addition of EH160 serves to condition the system to produce a chloride efflux in the presence of an external impermeable anion, as aspartate (Figure 5A, green trace).

Interestingly, in the presence of external impermeable anions, the time course of anionophore-driven chloride efflux favored by valinomycin is not exponential, but linear, as shown by the regression lines represented in blue in Figure 5; there, the correlation coefficient for a linear regression is *r* > 0.999, confirming the linearity of the traces. The exponential shape of the chloride efflux is due to the depletion of the anion from the LUV, that reduce the chloride gradient, and according to the Fick law, will reduce the anion flux. Conversely, the linear time course of the chloride efflux reflects a constant chloride gradient during the experiment.

This paradox may occur because, in the presence of the cation ionophore, the efflux of chloride driven by EH160 is accompanied with the facilitated efflux of potassium ions by valinomycin [or protons by cyanide-4-(trifluoromethoxy)phenylhydrazone, FCCP], thus maintaining the electro-neutrality of the process and resulting in a net solute loss from the LUV, with the consequent osmotic water withdrawal. The consequence is the maintenance of the concentration of the solutes, leaving essentially unaltered the ionic gradients.

**Figure 6:**
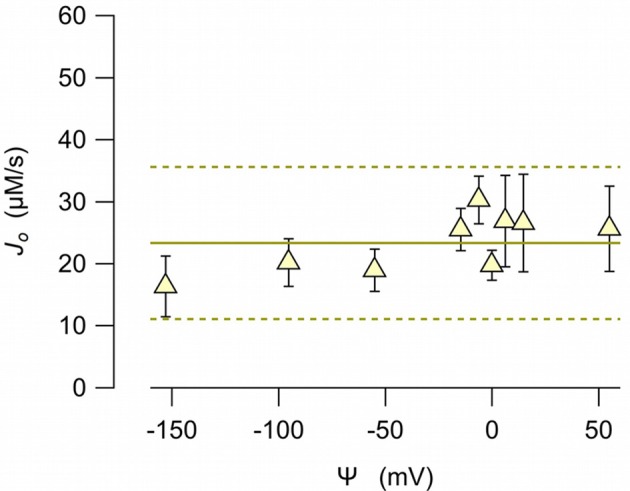
EH160-driven chloride initial efflux, *J*_0_, measured from LUV with 450 mM chloride in the internal solution, and 450 mM nitrate in the external solution. Different potassium concentrations at either side of the bilayer resulted in a potential difference, Ψ, by the presence of valinomycin (0.5 ng/ml). Symbols correspond to the mean values of, at least, three experiments, and the bars are the standard error of the mean. The continuous line is the average of all data, and the broken lines are the prediction limits with 95% confidence.

To examine whether the anionophore-driven transport is affected by the electric field, we measured the chloride efflux at different electric potential differences. To impose a membrane potential difference, we prepared LUV with different combinations of sodium chloride and potassium chloride inside, and sodium nitrate and potassium nitrate outside. The anion gradient was always the same (450 mM chloride inside, and 450 mM nitrate outside). Because valinomycin is permeable to potassium, but does not transport sodium, the bilayer potential difference, according to the Nernst equation, depends on the potassium concentration at both sides. The initial chloride efflux, *J*_0_, measured at potential differences between 50 to −150 mV (reference at the external side) resulted virtually independent from the voltage (Figure 6), indicating the EH160 activity is not voltage dependent.

### pH dependence of EH160 anionophore activity

**Figure 7:**
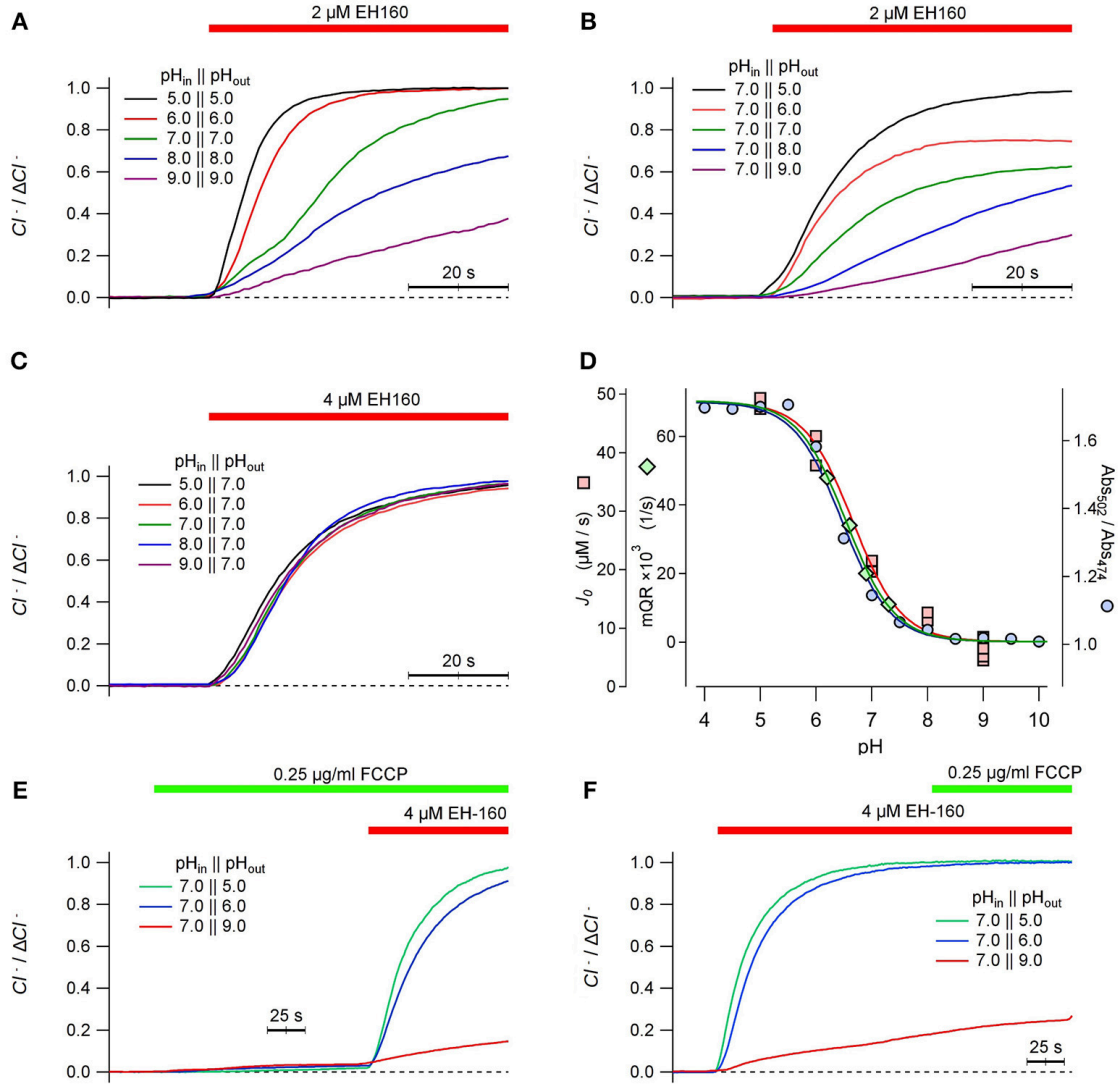
Time course of the external concentration of chloride induced by the anionophore EH-160 was measured in LUV with 450 mM internal chloride and 450 mM external ions, in symmetric pH buffer **(A)** and asymmetrical pH buffer, varying either the external pH **(B)** and the internal pH **(C)**, as indicated in the figure. The application of the anionophore is indicated by the horizontal bar. **(D)** The initial influx rate measured in LUV, *J*_0_, at different external pH is represented by squares; the maximum YFP-quenching rate, mQR, representing the iodide influx in FRT cells measured at different extracellular pH values, is pictured by diamonds. The ionic state of EH160, expressed as the ratios of absorbances at 502 and 474 nm (circles) is also plotted against the pH. The three continuous lines are the corresponding best fits of data with the Henderson-Hasselbach equation, normalized by the maximum and the minimum asymptotes. The time course of the external concentration of chloride measured in LUV treated with the proton ionophore FCCP prior the application of EH-160 **(E)**, or applying the proton ionophore FCCP after the induction of chloride efflux by the anionophore **(F)**, as shown by the horizontal bars.

We have previously reported that the transport efficacy of EH160 in LUV strongly depends on pH (Hernando et al., 2018). In LUV with the same values of pH in the internal and external buffers, the rate of the chloride efflux carried by EH160 is faster at acidic pH than at alkaline (Figure 7A). This pH dependency of the anionophore activity is similar when the internal pH in the LUV is kept constant at 7.0, and the external pH is varied from 5.0 to 9.0 (Figure 7B). Conversely, when the external pH is kept fix at 7.0, variations of the internal pH in LUV from 5.0 to 9.0 does not determine any significant variation of the anionophore induced chloride efflux (Figure 7C).

An interesting feature of these observations is that the chloride efflux seems to be independent of the proton gradient. If the anionophore co-transports anions and protons, in absence of an active transport mechanism, it is expected that the gradient of both ligands should determine the transport rate. On the contrary, the chloride efflux in absence of a pH gradient (Figure 7A) varies in the same manner as observed in the experiments where a pH gradient was imposed varying the external pH (Figure 7B). Moreover, imposing a pH gradient varying the internal LUV pH, but maintaining the external pH constant does not modify the chloride efflux (Figure 7C). We conclude that the anionophore chloride transport is independent of the pH gradient, but depends only on the external pH. These data is consistent with the influence of pH in the ionization state at which the anionophore is incorporated into the membrane, but does not affect the ionic transport itself. To test this hypothesis, we compared the pH dependency of the initial chloride efflux, *J*_0_, with the titration curve of the EH160. The titration curve was constructed plotting the ratio of absorbance measured at 502 and 474 nm of 20 μM EH160 against the pH of solvent buffer (Figure 7D, blue circles). These data were fitted with the Henderson–Hasselbach equation, yielding a pK_A_ of 6.47 ± 0.05. This titration curve can be superimposed with the plot of *J*_0_ vs. the external pH (Figure 7D, pink squares), confirming that the pH dependence of the anionophore-driven chloride efflux corresponds to the ionization state of EH160. Noteworthy, in mammalian cells the maximum quenching rate of the YFP fluorescence measured at different extracellular pH can be easily superimposed to the EH160 titration curve (Figure 7D, green diamonds), further confirming the similarity of the behavior of EH160 in plasma membranes and in model bilayers. Remarkably, the pK_A_ values estimated for the measurement of chloride efflux in LUV, 6.66 ± 0.07, and influx of iodide in cells, 6.54 ± 0.19, are not significantly different from the pK_A_ yielded from the EH160 titration.

To further reinforce the idea that anion transport is not coupled with proton (or hydroxide) transport, we measured the EH160 induced chloride efflux in LUV with different pH gradients, to observe the effect of the collapse of the gradient with the proton carrier FCCP. In these experiments, 0.25 μg/ml of FCCP were added to the external solution before the application of the anionophore (Figure 7E). The collapse of the pH gradient, induced putative proton transport induced by the FCCP, does not produce any significant chloride efflux; then, application of the anionophore induced a measurable chloride efflux corresponding to the external pH. Similarly, a chloride efflux, correspondent to the external pH, is observed upon application of EH160, but further application of FCCP does not modify the chloride efflux time course (Figure 7F). Application of FCCP will plausibly equilibrate the pH to values near to the external pH, thus dissipating the pH gradient. Similar results were obtained in experiments where the pH gradient was dissipated with nigericin, that is another proton transporter (data not shown). Thus, the lack of effect of this action on the chloride efflux rules out the coupling of anion transport and proton transport for EH160, confirming the independence between the anion transport and the proton gradient present.

### Anionophore-driven transport in mammalian cells

**Figure 8:**
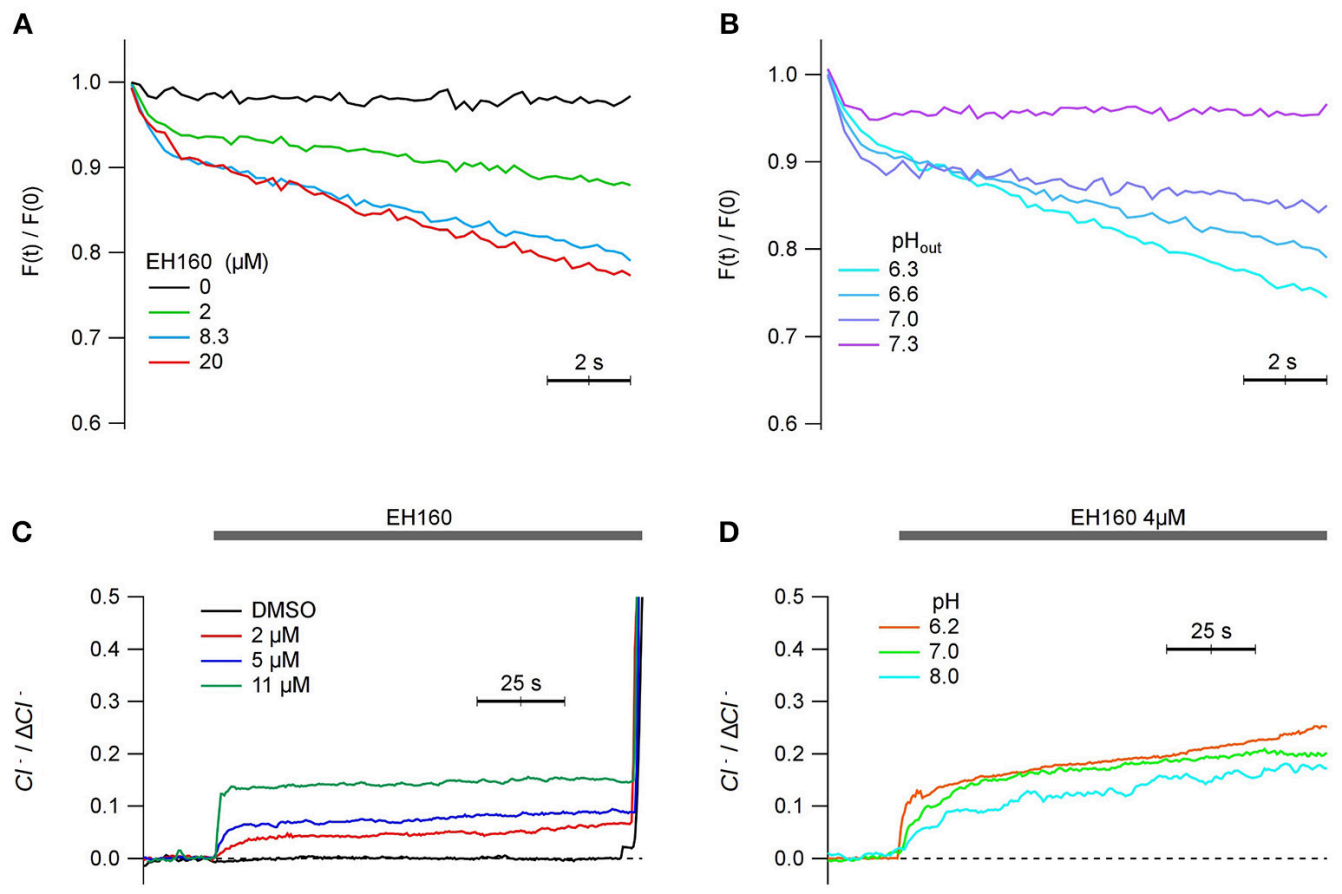
Anionophore-driven transport in mammalian cells. **(A,B)** show the time course of the iodide-sensitive YFP fluorescence, normalized by the initial fluorescence [F(t)/F(0)], measured in FRT cells after the addition of 100 mM iodide in the external solution. The decrease of the fluorescence, due to the quenching of YFP fluorescence by iodide, represents the influx of the halide. Each trace is the average of three measurements. **(A)** Traces obtained with the treatment of cells with different concentrations of EH160, at pH 6.6. **(B)** Different efficacy of 8.2 μM EH160 at different extracellular pH. **(C,D)** represents the time course of the external concentration of chloride on HEK cells perfused with different EH160 concentrations **(C)** and at different pH **(D)**. Data was normalized by the maximum anion change, Δ*Cl*^−^.

Since the ultimate objective of the characterization and further optimization of the anionophores is to open the possibility to use them in cells as therapeutic agents, we report the proof of concept that this class of carriers is able to transport anions in mammalian cells. Therefore, we have repeated the measurements of anionophore-driven iodine influx and chloride efflux in mammalian cells. The toxicity of EH-160 (TD_50_ = 7.1 ± 1.1 μM) is reduced respect to that of prodigiosin (TD_50_ = 2.9 ± 2.4 μM). Figure 8A shows the time course of the iodide quenching of the YFP fluorescence, representing the influx of the halide into the FRT cells. The iodide influx is clearly dependent of the concentration of EH160. Similarly, a chloride efflux was observed in HEK cells upon the application of the anionophore at different concentrations (Figure 8C). Quantification of the chloride efflux under these conditions was difficult because the removal of chloride from the external solution modifies the membrane potential and activates endogenous mechanism to maintain the cell homoeostasis that partially hidden the signal of anionophore-driven transport. It is, however, clear that EH160 induces halide transport in living mammalian cells.

Interestingly, the transport capacity of the anionophore also depends on the extracellular pH. Using both methods to display the anion transport in cells, the fluorescent probe to measure the iodide influx (Figure 8B), and the use of ISE to evaluate the chloride efflux (Figure 8D), we observed that the efficacy of transport increases as the extracellular pH is more acidic, similarly as observed for the measurements in LUV. We conclude that the transport properties of the triazole derivative of prodigiosine in mammalian cells are similar to those observed in LUV.

## Discussion

We have examined the properties of a prodigiosin-inspired anionophore to characterize its anion transport properties. A detectable chloride efflux was measured in LUV for the 1,2,3-triazole heterocycle assayed, EH160 at micromolar concentrations (Figure 1A). At equimolar concentrations, the natural product prodigiosin showed a considerable higher potency than EH160. Notably, the application of the anionophore solvent, DMSO, does not induce any chloride efflux in LUV (Figure 1A), confirming that the results reflect the anion transport driven by the anionophore. The dose-response curves for anionophores shown in Figure 2 further confirm that the examined molecules are responsible for the observed chloride efflux. These data indicated that EH160 in LUV exerts the half of its maximum activity at 5.6 μM (Figure 2B). This value is two orders of magnitude higher than that we reported before (Hernando et al., 2018). This discrepancy is due to the different method employed to calculate this EC_50_ value. In our previous work the dose response curves were empirically constructed plotting the amount of chloride transported after a time interval (300 s). This is an arbitrary time interval and this measurement is useful to compare the potency of series of compounds. Here we have obtained this EC_50_ value fitting the initial chloride flux calculated according to Equations (1, 2). This is thus an absolute measurement of the potency of the compound in the assayed conditions. The main advantage of the triazole derivatives is that they exhibit a reduced toxicity while retaining a remarkably high transport activity. Prodigiosine is a highly toxic compound (TD_50_ = 2.9 μM), as reported in numerous studies (Manderville, 2001). Although it is likely that cytotoxicity account for some of the intriguing pharmacological properties of this compound it jeopardizes its potential application as CFTR replacement therapy. The TD_50_ of EH160 is 7.1 μM, that is, indeed, no very different of the EC_50_ of 5.4 μM. Although the difference is small, it should be possible to optimize the anionophores to obtain less toxic substances. In any case, to apply these transporters for therapeutic uses, one have to consider also the efficacy of the anionophores. The efficacy (maximum quenching rate) of the iodide influx driven by the anionophore EH160 is similar to the CFTR activated by applying 20 μM forskolin and 10 μM genistein in FRT cells (Hernando et al., 2018). Since the CFTR activity of the CFTR transfected in the FRT cells is more than 13-fold greater that expected in human bronchial cells (Taddei et al., 2004; Moran et al., 2005; Kreindler et al., 2009; Melani et al., 2010; Gianotti et al., 2013, 2016). Thus, to induce an anion transport equivalent to that expected in bronchial cells, it would be necessary to apply <0.5 μM of EH160, significantly increasing the width of the therapeutic window.

Experiments were done with a very simple system, unilamellar vesicles, to avoid the contribution of other anion transport mechanisms present in cells. Another advantage to use such artificial system is the possibility to create large anion gradients, up to 450 mM, that improves the resolution of the measurements. Noteworthy, the phospholipid composition seems not to modify significantly the transport capacity of the anionophores. We cannot exclude, however, that mayor modifications in surface charge or membrane viscosity could induce differences on chloride efflux. We have also assayed the two most biological relevant cations, sodium and potassium, and could not find any difference on chloride efflux. Thus, we concluded that these cations very unlikely contribute to anionophore-driven chloride transport.

**Figure 9:**
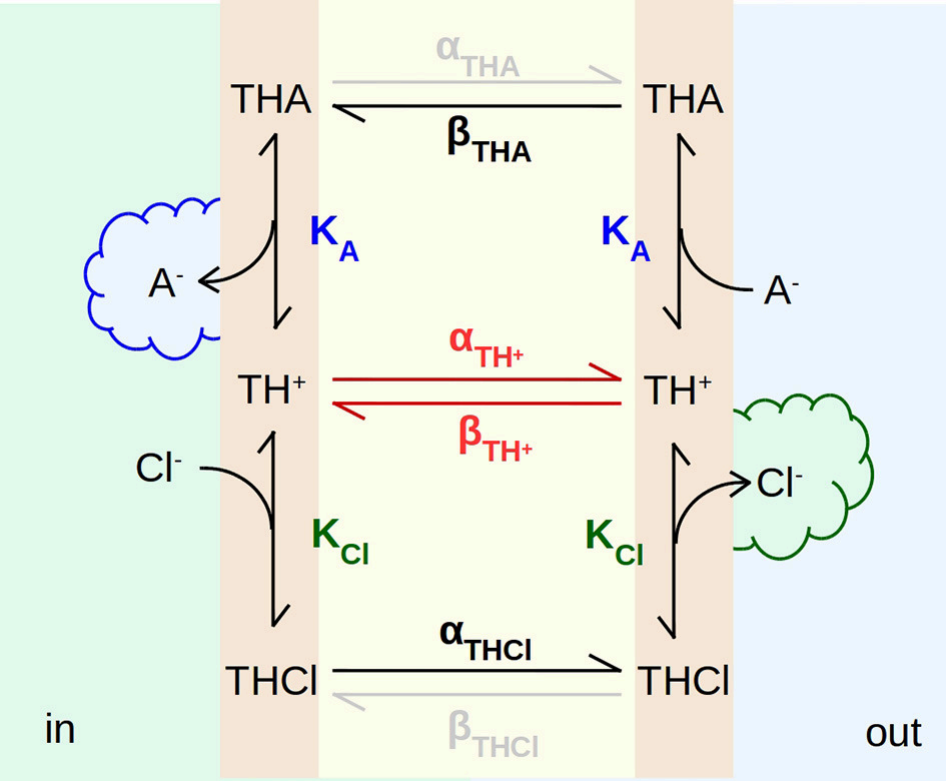
Scheme of the mechanism of transport of anions across a bilayer driven by the anionophore EH160. **TH**^+^, **TCl**, and **TA** are anion-free, chloride-bound, and any other anion-bound forms of the EH160 transporter, respectively. These three carrier forms are located in the aqueous solution-bilayer interface, where they can reversibly bind anions, with a binding equilibrium constant, **K**_**Cl**_ and **K**_**A**_, for chloride and the second anion, respectively. Each anionophore form can diffuse across the hydrophobic region of the bilayer with a rate constant **α** and **β**. The high chloride concentration at the internal side **(in)**, and the high concentration of a second permeable anion (nitrate or bicarbonate) at the outer side **(out)**, will produce a chloride efflux.

To better explain the EH160-driven transport we propose the model presented in Figure 9, where the carrier incorporated in the bilayer has three states, the free carrier, **TH**^+^, the carrier bound to chloride, **THCl**, and the carrier bound to a second anion, **THA**, for example nitrate, bicarbonate or gluconate. The binding to the carrier occurs in the aqueous solution-bilayer interface with the first order equilibrium constants **K**_**Cl**_ and **K**_**A**_ for chloride and the second ion, respectively. The internal solution (**in**) has a high concentration of chloride and there is no second anion; on the other hand, the concentration of the second anion is high in the external solution (**out**), meanwhile chloride is virtually absent. Thus, when the anionophore is on the internal face of the bilayer, the binding of chloride and the release of the second anion are favored, while the unbinding of chloride and the binding of the second anion takes place on the outer face of the bilayer. In this manner, as the anionophore diffuses across the bilayer, a chloride efflux, as a result of the exchange with the second anion, is established.

These three anionophore forms diffuse across the hydrocarbon chains with rates of α and β. If we assume that the bilayer is symmetric and homogeneous, for a given carrier state, rate constants α and β are equivalent. However, the diffusion of the carrier will be determined by the binding state of the anionophore significantly. The diffusion rates for the free carrier, **TH**^+^, are probably lower than for the chloride bound form, **THCl**, or the **THA** form where **A**^−^ is a permeable anion as bicarbonate or nitrate. It is possible to speculate that the energy barrier to cross the hydrophobic region of the membrane is higher for the **TH**^+^ form, bearing a the positive net charge (see Figure 1), than the complexes **THCl** and **THA**. Complexed anions form a tightly bound ion par with the carrier which is overall neutral, and undergoes the diffusion across the hydrophobic environment easily. Thus, the permeability of different anions will depend on both, the binding equilibrium constant, and the diffusion rate of the anion-EH160 complex.

Comparing with permeable ions (chloride, nitrate, bicarbonate), the impermeable anions, such as sulfate, phosphate or gluconate, are characterized by a more stable interaction with water, with a more negative hydration enthalpy and Gibbs energy (Marcus, 1987, 1991). The structural study and theoretical analysis of synthetic prodigiosines have shown that anions bind the molecule in a groove formed by the three N–H groups (Díaz de Greñu et al., 2011; García-Valverde et al., 2012; Hernando et al., 2018). A similar binding of anions was also described for analogs of the marine alkaloids tambjamines (Iglesias Hernández et al., 2012; Hernando et al., 2014). Accordingly, the difficulty to remove the hydration water from these impermeable anions, and their larger molecular volumes (Marcus, 1993) will contribute to reduce the affinity of these anions for the carrier, and therefore the form **THA** is virtually absent, abolishing the flux of these anions across the bilayer. However, as long as electroneutrality conditions are maintained, shunting the bilayer with a cation carrier, the chloride flux is appropriately restored (Figure 5). Albeit, the chloride efflux occurring in absence of counter-anion influx (see Figure 5), when the **THA** state is not present, demonstrates that the free, unbounded anionophore **TH**^+^, is able to diffuse across the bilayer. At this point we could delineate the transport mechanism of EH160 as a molecular carrier embedded in the lipid bilayer. It should bind an anion at the bilayer interface at one side, cross the bilayer, and release the anion at the other side; the same mechanism occurs when the second ion is transported in the opposite direction, closing the transport cycle.

Interestingly, in the presence of external impermeable anions, the time course of anionophore-driven chloride efflux favored by valinomycin is not exponential, as described by equation 1, but becomes linear (Figures 5B,C). The exponential shape of the chloride efflux curve is due to the depletion of the anion from the LUV, that reduces the chloride gradient. According to the Fick law, this results in the reduction of the anion flux (observed as the derivative of the time course). Hypothetically the linear time course of the chloride efflux is due to a shrinking volume of LUV, as the efflux of a chloride ion should be accompanied with the efflux of a potassium ion mediated by valinomycin (or protons through FCCP), to maintain the electroneutrality. The result should be a net solute lost, with the consequent water osmotic withdrawal. These movements, within the measuring time interval, would result in the maintenance of the concentration of the solutes, and the consequent maintenance of the ionic gradients.

In the model depicted in Figure 9 the movement of anions facilitated by the EH160 carrier has been illustrated. According to this scheme, EH160 is a reversible uniporter, and the direction of the flux is driven by the chemical gradient. Conversely, due to the fact that the measurements of the effect of prodigiosine and its derivatives in cells have shown that they induce cellular death accompanied with modifications of intracellular pH levels (Ohkuma et al., 1998; Castillo-Avila et al., 2005; Seganish and Davis, 2005; Díaz de Greñu et al., 2011; Gale et al., 2013; Cheung et al., 2018), it has been suggested that this class of anionophores are H^+^/Cl^−^-symporters. However, we have seen that the pH influence of the EH160-transport activity is independent of the H^+^ gradient (Figures 7, 8), but just on the ionization state of the carrier (Figure 7D). Indeed, there is no modification of the chloride efflux when the proton gradient is dissipated using proton carriers like FCCP (Figures 7E,F) or nigericin (data not shown). This is consistent with the conformational analysis showing that the anion binding is favored by the protonated form of a model prodigiosine (García-Valverde et al., 2012). Therefore, amount of an active protonated carrier in the bilayer will be determined by the pH in the external solution, which has a several orders of magnitude larger volume, and will dominate the equilibria in the three-compartment system formed by the internal space, the external space and the bilayer.

To estimate the chloride exchange rate in LUV we have to take into account the concentration of the anionophore in the bilayer membrane based on the concentration of EH160 in the aqueous solution. The average radius of the LUV, 47 nm, and the bilayer thickness of 3.75 nm, were obtained by small angle x-ray scattering (Baroni et al., 2014). It follows that the bilayer volume per vesicle is 3.39 × 10^−16^ cm^3^. From the final external chloride concentration, ΔCl ≈ 0.9 mM, and the total volume of the assay, 3.5 cm^3^ we can estimate the total number of vesicles 1.61 × 10^13^, and the total bilayer volume in the sample, 1.55 × 10^−3^ cm^3^. To estimate the concentration of the anionophore in the bilayer, we used the water/*n*-octanol partition coefficient as calculated by the computational chemistry suite Marvin Sketch (https://www.chemaxon.com). It allows to calculate the partition coefficient of ionized and non-ionized species from the molecular structure (Viswanadhan et al., 1989), taking into account the ionization at a given pH, and the effect of the counter ion concentration. Thus, the *n*-octanol/buffer partition coefficient for EH160 in 450 mM NaCl (or KCl) at pH 7.5 is *P*_*octanol*/*buffer*_ = 68.1 (logP = 1.83). Hence, for an anionophore concentration of 1 μM, we expect a bilayer anionophore concentration of 6.62 × 10^−8^ moles cm^3^, that corresponds to 6.18 × 10^13^ molecules of EH160 in the bilayers. The initial chloride efflux expected for 1 μM EH160 is 1.31 × 10^−5^ M/s, that corresponds to a transport rate of 7.45 × 10^−22^ moles of chloride/s per anionophore molecule, that represents 449 chloride ions/s per anionophore molecule. It is important to highlight that the efflux, and consequently the transport rate, depends on the chloride gradient, that for LUV experiments is ~450 mM. In contrast, in mammalian cells, we could assume that the chloride gradient is ~120 mM (≥30 mM intracellular and 150 mM extracellular), therefore, the transport rate should be scaled accordingly, resulting in 120 chloride ions/s per anionophore molecule. These values are similar to the exchange rates reported for other natural ion carriers in membranes, like the bacterial sugar transporters 2 × 10^2^ (Waygood and Steeves, 1980), or the sodium calcium exchangers 5 × 10^3^ (Baazov et al., 1999), but significantly lower than those characteristic of ion channel transport (6 × 10^6^-12 × 10^7^; Hille, 2001). A detailed calculation of the chloride turnover is presented as Supplementary Material.

We assayed the anionophores in a cellular model to assess whether anionophores could transport halides across the plasma membrane. By using an iodide-sensitive YFP to monitor the intracellular iodide concentration, we could demonstrate that, as described for other small organic molecules such as calix[4]pyrroles (Ko et al., 2014), tambjamines (Soto-Cerrato et al., 2015), ortho-phenylene bis-ureas (Dias et al., 2018), and bis-(p-nitrophenyl)ureidodecalins (Li et al., 2016), EH160 is able to transport this ion through the cell membrane (Figures 8A,B). The advantage of the iodide influx measurements is that the ion gradient driving the flux is well-controlled in the experiment. On the other hand, measurements of the chloride efflux revealed difficult because cells regulate the intracellular chloride concentration maintaining it low: in epithelium chloride concentration is ≤ 30 mM; in an experiment, when the extracellular chloride is removed, the effective gradient is 15-fold smaller that used in LUV experiments, and the efflux must be proportionally smaller. On the other hand, the cell homoeostasis implies a series of mechanisms that transport different ions, including several chloride and bicarbonate transporters, that may conceal a proper estimation of the anionophore- driven chloride efflux. Nevertheless, we could demonstrate that anionophores do induce chloride efflux in mammalian cells, with general characteristics similar to those observed in LUV bilayers (Figures 8C,D). Chloride efflux was observed when substituting the extracellular chloride by nitrate.

Here we have complemented the data previously reported (Hernando et al., 2018), designing a series of experiments useful to understand the transport mechanism of triazol derivatives of prodigiosine. These experiments demonstrate that these anionophores could be used to promote chloride and bicarbonate transport in cells, i.e., are good candidates to replace the defective or missing CFTR in an attempt to design a new cystic fibrosis therapy, as proposed for other anion transporters (Shen et al., 2012; Valkenier et al., 2014; Li et al., 2016, 2017; Liu et al., 2016; Dias et al., 2018). The analysis of anionophore-induced anion transport in cells needs, in any case, to be extended, studying the anionophore-induced ion transport in epithelial models, where the polarization of cells plays a fundamental role on the directionality of ion transport, to find the best suited compounds to become candidates for cystic fibrosis therapy. Preliminary experiments on other prodigiosine and tambjamine derivatives have shown that the properties of the anionophore EH160 could be extended to other analogous compounds, opening the possibility to design molecules optimized for clinical development. This proof of concept represents an encouraging promise for future developments toward a mutant-independent cystic fibrosis therapy.

## Author contributions

OM and RQ planned the study. OM, RQ, and EC designed the experiments and analyzed data. RQ and MG-V synthesized the anionophores. CC performed the experiment on vesicles under the supervision of OM. VC, DB, and MF performed the experiments on cells under the supervision of OM and EC. OM and RQ wrote the manuscript.

### Conflict of interest statement

The authors declare that the research was conducted in the absence of any commercial or financial relationships that could be construed as a potential conflict of interest.
