# Supplementary material for: Anion-Transport Mechanism of a Triazole-Bearing Derivative of Prodigiosine: A Candidate for Cystic Fibrosis Therapy
Source: Front Pharmacol. 2018 Aug 7;9:852. doi: 10.3389/fphar.2018.00852 (PMC6090297; doi:10.3389/fphar.2018.00852)
Supplement: Supplementary file 1 [file Data_Sheet_1.PDF]

Anion-transport mechanism of a triazole- bearing derivative of prodigiosine: a candidate for cystic fibrosis therapy

Claudia Cossu[1], Michele Fiore[1], Debora Baroni[1], Valeria Capurro[2], Emanuela Caci[2], Maria Garcia-Valverde[3], Roberto Quesada[3] and Oscar Moran[1*]

[1]Istituto di Biofisica, CNR, Genova, Italy;
[2]U.O.C. Genetica Medica, Istituto Giannina Gaslini, Genova, Italy
[3]Departamento de Química, Facultad de Ciencias, Universidad de Burgos, Burgos, Spain

Supplementary material

Calculation of the chloride turnover per EH160 molecule

The total volume of LUV, was estimated from the dilution of the intra-vesicle initial chloride concentration Cl_i = 0.45 M in the total preparation volume $V_t = V_o + V_v$, where V_v is the total vesicle volume, the outer buffer volumes V_o = 3.5 cm^3, and the final chloride concentration after the LUV lysis by detergent, ΔCl = 9×10^{-4} M:

$$V_v = \frac{V_o \times \Delta Cl}{Cl_i - \Delta Cl} \quad ,$$

yielding V_v= 7.01×10^{-3} cm^3, and Vt = 3.0701×10^{-3} cm^3.

The average radius of the LUV extruded through a single 100 nm mesh filter is R_v = 4.7×10^{-6} cm, and the bilayer thickness L_b = 3.75×10^{-7} cm, as measured by small angle x-ray scattering for the same LUV preparation (Baroni et al., 2014). Thus, assuming that LUV are spherical, the total LUV volume is V_u = 4.35×10^{-16} cm^3, the internal LUV volume is V_i = 3.39×10^{-16} cm^3, and the bilayer volume is $V_b = V_u - V_i$ = 9.6×10^{-17} cm^3. Therefore, the total number of vesicles, n_v, is:

$$n_v = \frac{V_v}{V_u} = 1.61 \times 10^{13} \quad ,$$

and the total bilayer volume is $V_{tb} = V_b \times n_v$ = 1.55×10^{-3} cm^3.

To calculate the anioniophore concentration in the lipid phase we used the octanol/water partition coefficient of the ionised species of EH160, as estimated by the computational chemistry suite Marvin Sketch (https://www.chemaxon.com) (Viswanadhan et al., 1989), P = 68.1. Therefore, the concentration of EH160 in the lipid fraction is:

$$A_b = \frac{P\, A_o V_t}{V_o + V_{tb} P} = 6.62 \times 10^{-8} \text{moles/cm}^3 \quad ,$$

and the number of moles of EH160 in the bilayer is $M_b = A_b \times V_{tb}$ = 1.03×10^{-10}. Multiplying M_b to the Avogadro number, $\mathcal{N}$=6.026×10^{23} moles^{-1}, we obtain the number of EH160 molecules in the bilayer, N_b = 6.18×10^{13}.

We calculate the chloride turnover for EH160 molecule for our standard vesicle preparation where we added the LUV, and with an anionophore concentration of 1 µM, A_o = 1×10^{-9} moles/cm^3. Considering the maximum chloride efflux J_{max}= 8.73×10^{-8} moles/(cm^3×s), and the EC$_{50}$ = 5.64 µM = 5.64×10^{-9} moles/cm^3, the expected initial chloride efflux is:

$$J_0 = J_{max} \frac{A_o}{A_o + \text{EC}_{50}} = 1.31 \times 10^{-8} \text{moles /(cm}^3 \cdot \text{s)}$$

Thus, the net chloride efflux is $J'_0 = J_0 \times V_b$ = 4.6×10^{-8} moles/s, and the chloride efflux per EH160 molecule is $j = J'_0 / N_b$ = 7.45×10^{-22} moles/s. Multiplying j by $\mathcal{N}$ we obtain a turnover rate of ~449 chloride ions / s per EG160 molecule.
